# Effects of an optimised POCT guided diagnostic and treatment strategy for symptoms of uncomplicated UTI on use of appropriate antibiotics and uptake into primary care practice

**DOI:** 10.1186/1745-6215-14-S1-P10

**Published:** 2013-11-29

**Authors:** Janine Bates, Emma Thomas-Jones, Nigel Kirby, Tim Pickles, Rhys Thomas, Emily Bongard, Micaela Gal, Paul Little, Theo Verheij, Carlos Llor, David Cohen, Nick Francis, Kerry Hood, Christopher Butler

**Affiliations:** 1 Cardiff University, Cardiff, UK; 2 Julius Center for Health Sciences and Primary Care, Utrecht, The Netherlands; 3 Primary Care Centre Jaume I University Rovira i Virgili, Tarragona, Spain; 4 University of Southampton Aldermoor Health Centre, Southampton, UK; 5 University of South Wales, Pontypridd, UK

## 

Patients presenting with UTI symptoms are among the most frequent clinical presentations in primary care accounting for 15% of all community antibiotic prescriptions. The diagnostic accuracy of clinical assessment and the most appropriate management of uncomplicated UTI remain unclear. Primary care clinicians are more likely to treat empirically without additional testing.

Some advances are being made in the development of novel Point of Care Tests (POCTs ) for diagnosis of infection and antibiotic resistance in primary care. The incorporation of a test, which enumerates and identifies pathogens and their antibiotic resistance, into a management strategy for UTI has great potential for improving patient outcomes reducing antibiotic use and in turn in reducing the incidence of antimicrobial resistance.

While some new promising POCT technologies are being developed, these have not been fully evaluated in primary care. Robust evaluation, providing solid evidence supporting POCT use, which is based on patient benefit, cost-effectiveness, therapeutic choice, microbiological outcomes, and uptake and acceptance into primary care is required.

We have designed a ‘process-pathway' model which incorporates acceptability parameters for a POCT in primary care, and incorporates four distinct stages:

Stage 1. Piloting of a novel POCT for diagnosis of UTI in primary care.

Stage 2. An observational stage to describe current primary care management of uncomplicated UTI and incidence of uropathogens

Stage 3. A randomised controlled trial rigorously evaluating the POCT as part of a rapid diagnostic and treatment regime for uncomplicated UTI.

Stage 4. Evaluating the uptake of the POCT in general practice.

